# Effects of ethnic density on the risk of compulsory psychiatric admission for individuals attending secondary care mental health services: evidence from a large-scale study in England

**DOI:** 10.1017/S0033291721001768

**Published:** 2023-01

**Authors:** Orla McBride, Craig Duncan, Liz Twigg, Patrick Keown, Kamaldeep Bhui, Jan Scott, Helen Parsons, David Crepaz-Keay, Eva Cyhlarova, Scott Weich

**Affiliations:** 1 Ulster University, Coleraine, UK; 2 University of Portsmouth, Portsmouth, UK; 3 Academic Psychiatry Campus for Ageing & Vitality, Newcastle University, Newcastle upon Tyne, UK; 4 Department of Psychiatry, University of Oxford, Oxford, UK; 5 The World Psychiatric Associations UK Collaborating Centre, London, UK; 6 Institute of Neuroscience, Newcastle University, Newcastle upon Tyne, UK; 7 Warwick Medical School Clinical Trials Unit, University of Warwick, Coventry, UK; 8 Mental Health Foundation, London, UK; 9 London School of Economics and Political Science, London, UK; 10 School of Health and Related Research, University of Sheffield, Sheffield, UK

**Keywords:** Compulsory admission, deprivation, ethnic density, ethnicity, mental health act

## Abstract

**Background:**

Black, Asian and minority ethnicity groups may experience better health outcomes when living in areas of high own-group ethnic density – the so-called ‘ethnic density’ hypothesis. We tested this hypothesis for the treatment outcome of compulsory admission.

**Methods:**

Data from the 2010–2011 Mental Health Minimum Dataset (*N* = 1 053 617) was linked to the 2011 Census and 2010 Index of Multiple Deprivation. Own-group ethnic density was calculated by dividing the number of residents per ethnic group for each lower layer super output area (LSOA) in the Census by the LSOA total population. Multilevel modelling estimated the effect of own-group ethnic density on the risk of compulsory admission by ethnic group (White British, White other, Black, Asian and mixed), accounting for patient characteristics (age and gender), area-level deprivation and population density.

**Results:**

Asian and White British patients experienced a reduced risk of compulsory admission when living in the areas of high own-group ethnic density [odds ratios (OR) 0.97, 95% credible interval (CI) 0.95–0.99 and 0.94, 95% CI 0.93–0.95, respectively], whereas White minority patients were at increased risk when living in neighbourhoods of higher own-group ethnic concentration (OR 1.18, 95% CI 1.11–1.26). Higher levels of own-group ethnic density were associated with an increased risk of compulsory admission for mixed-ethnicity patients, but only when deprivation and population density were excluded from the model. Neighbourhood-level concentration of own-group ethnicity for Black patients did not influence the risk of compulsory admission.

**Conclusions:**

We found only minimal support for the ethnic density hypothesis for the treatment outcome of compulsory admission to under the Mental Health Act.

## Introduction

The prevalence of psychiatric disorders, and in particular psychotic disorders, is considerably higher (~1.5–3 times) among ethnic minority groups living in the UK compared to the White British majority (Kirkbride et al., 2012; Morgan, Knowles, & Hutchinson, 2019; Qassem et al., 2015; Weich et al., 2004). Identifying plausible causal environmental pathways to help explain, and ultimately reduce, these substantial ethnic health inequalities is an important, yet complex, area of investigation (Das-Munshi, Becares, Dewey, Stansfeld, & Prince, 2010). Given that individuals of minority ethnic status in the UK have traditionally resided in clusters in urban areas (Finney, 2013), investigating the characteristics of such places, namely area-level deprivation, urbanicity and ethnic density, might give rise to better explanations of how negative mental health outcomes among minority ethnic groups emerge (Becares & Nazroo, 2015; Karlsen, Nazroo, & Stephenson, 2002).

Living in urban areas typically characterised by high levels of socio-economic deprivation, over-crowding and low-quality housing may not be conducive to mental wellbeing, particularly if protective factors such as social support are weak (Lee et al., 2020). Despite the potentially detrimental impact of this type of living environment on mental health and wellbeing, some characteristics of these residential environments (e.g. sharing similar cultural, linguistic and religious values with neighbours) may buffer the negative impacts of social disadvantage in these minority groups – the so-called ‘ethnic density’ effect (Halpern & Nazroo, 2000; Pickett & Wilkinson, 2008). From this perspective, it is the level of ethnic concentration in the area in which a person lives that matters most in influencing the individual's risk of experience mental ill-health (Morgan & Hutchinson, 2010). For example, Schofield, Ashworth, and Jones (2011) revealed a negative association between the prevalence of psychotic disorders and the size of the local ethnic group relative to the total population, meaning that individuals of ethnic minority status experienced a substantial increased risk of developing psychosis compared to their White counterparts, but only when they lived in the areas of low ethnic concentration.

That area-level ethnic concentration might play an important role in influencing the onset of mental disorders may have important implications for attempting to explain the persistent ethnic inequalities in mental health treatment outcomes, such as compulsory psychiatric admission under the Mental Health Act (MHA). It could be argued that compulsory admission is not an optimal treatment outcome but rather a necessary experience along the mental health care pathway if other options are not available in crisis. The use of compulsory admission is often considered necessary to reduce the risk of immediate and/or serious physical harm to the patient themselves or others. The deprivation of liberty under the MHA is a serious clinical intervention, and one that often sits uneasily with clinicians, service planners and policy makers. Compulsory detention can also be a very negative and distressing experience for services users and their families (Akther et al., 2019; Mann et al., 2014). Despite this unease, evidence from administrative data indicates that rates of compulsory admission have risen steadily in England in recent years (Health and Social Care Information Centre, 2015). Individuals of Black, Asian and minority ethnicity groups experience higher rates of compulsory admission compared to their White counterparts, and have been disproportionately affected by these rising rates (Singh, Greenwood, White, & Churchill, 2007; Weich et al., 2017). For example, in 2018–2019, although the total rate of detention under the MHA was 94.3 per 100 000 population, this varied across ethnic groups: 74.4 for White, 138.2 for mixed-ethnicity, 93.4 for Asian or Asian British and 278.2 for Black or Black British (NHS Digital, 2019). Tackling these profound inequalities in compulsory psychiatric treatment is a key priority for the UK Government following the 2018 Independent Review of the Mental Health Act (Department of Health and Social Care, 2018).

A recent systematic and meta-analysis review revealed that a substantial body of research investigating the excess risk of compulsory detention among ethnic minorities (48% of studies included in the review) offered no explanation for the variation in the risk of detention among minority groups, or proposed tentative explanations [e.g. (i) differences in experiences of symptoms of mental disorders; (ii) variations in self-management of such symptoms; (iii) varied care pathways into mental health services and (iv) being subjected to different professional practices by those involved in mental health treatment delivery] without support from primary evidence (Barnett et al., 2019).

Classic studies, such as that conducted by Faris and Dunham (1939), have long hinted at the plausibility of the ethnic density effect in relation to the risk of compulsory psychiatric admission. Wechsler and Pugh (1967) argued that ‘people who do not “fit” in a community should have higher rates [of psychiatric hospital admission] than those who do’ (p. 220). Previous research in this area in the UK has produced mixed findings, however. Over two decades ago, Cochrane and Bal (1988) demonstrated that, with the exception of Irish-born males, ethnic density (measured by proxy using country of birth) at the regional health authority level in England was unrelated to the rates of hospital admissions for schizophrenia. However, Keown et al.'s (2016) analysis of NHS mental health admission rate data from 2005/06 revealed that, running somewhat counter to the ethnic density effect hypothesis, minority ethnic groups with low levels of clustering at the local authority spatial level experienced low rates of compulsory admission. Based on an ecological analysis of patterns of compulsory admission across England, Keown et al. (2016) demonstrated that area-level ethnic density (measured at the Primary Care Trust level with population sizes of about 350 000) was strongly associated with the rate of compulsory in-patient treatment, but only in urban areas where there are most pressures on services, less capacity per head of population and also the greatest concentration of risk factors for psychosis. However, it may be that the protective effect of ethnic density largely operates at lower spatial levels (e.g. neighbourhood level), and so was not observable at higher spatial levels.

Clearly, more robust research is required to explain the substantial variation in the risk of detention if this trend is to be halted and reversed (Mann et al., 2014). For example, if it can be demonstrated that living in the areas of high own-group ethnic density is protective against compulsory admission, either for all or specific minority ethnic groups, then service commissioners and providers could use this information to target intervention efforts towards improving social inclusion for minority ethnic groups. Alternatively, if living in the areas of high own-group ethnic density increases the risk of compulsory admission, either uniformly or for specific ethnic minority groups, then we must determine significant factors (e.g. cultural issues, language difficulties, etc.) which may be hampering appropriate engagement with the mental health care system.

Here, we attempt to advance the evidence base by analysing data from the Mental Health Minimum Dataset (MHMDS), the mandatory administrative dataset for providers of secondary care mental health services to ~1.2 million patients in England during 2010–2011. The study had two aims: (1) to test the effect of living in an area of high own-group ethnic density on risk of compulsory admission; and (2) to assess whether area-level deprivation and population density were potential confounders of this effect. Consistent with the ‘ethnic density’ hypothesis, we hypothesised that living in the areas of high own-group ethnic density would produce a protective effect against compulsory admission, which might differ across ethnic groups, but that the effect would remain (or even strengthen) after adjusting for the area-level deprivation and population density.

## Method

### Primary data sources and study population

The 2010–2011 MHMDS includes data on all adults aged 18 years or older (and a small number of individuals aged under 18 years) who have received specialist adult mental health care services in a secondary care setting between 1 April 2010 and 31 March 2011. The MHMDS was set-up in 2007 and was designed to support the legal requirement of the NHS to administer and apply the MHA. Although the quality of mandatory returns by NHS Provider Trusts improves annually, quality checks were conducted by our team prior to undertaking statistical analyses to screen the 2010–2011 MHMDS for possible errors and omissions. Data from eight NHS Provider Trusts were ultimately excluded because: (1) three independent providers had no spatial identifiers, which prevented linkage (see next section); (2) four NHS Trusts had no data on patients' legal status under the MHA, which prevented determination of study outcome for patients and (3) one NHS Trust had no inpatient beds, which meant that patients in that Trust could not be compulsorily detained under the MHA. The final study sample consisted of 1 238 188 patients who received care from 64 NHS Provider Trusts.

### Linkage to secondary data sources

Spatial identifiers on patient records allowed linkage to demographic data (ethnicity and population estimates) based on the 2011 Census (Office of National Statistics, 2012) and to the 2010 Index of Multiple Deprivation (IMD) (Department for Communities and Local Government, 2011) at the level of Lower Layer Super Output Areas (LSOAs). There are 32 844 LSOAs in England and they are primarily used for the reporting of UK Census data and have an average population of 1614 persons. Service setting identifiers meant the Provider Trusts in which patients received care could also be considered in statistical models (Weich et al., 2017).

### Outcome

The main outcome variable was compulsory admission to hospital under the MHA during the 2010–2011 reporting period, compared with all other types of care (including voluntary hospital admission and community-based care only). Given that no single variable in the 2010/2011 MHMDS described the study outcome, it was derived from several variables including admissions and discharges, bed days, receiving community treatment and legal detention status under the MHA. It was possible to identify whether patients had been, during the reporting period, compulsorily admitted to hospital and the highest level of legal restriction (according to the MHA) recorded on their care record, but not the number or duration of compulsory admissions. Patients were defined as experiencing a compulsory admission if they had been detained in hospital at any point during the reporting period under Sections 2, 3, 4, 35, 36, 37, 38, 47 and 48 of the 2007 MHA (approximately 95% of individuals admitted to hospital under the MHA). We excluded patients detained under sections of the MHA concerned only with conveyance to, or assessment in, a place of safety (Sections 135 and 136) as these do not in themselves necessarily mean that the person will be admitted to a psychiatric ward. Moreover, there are on-going concerns about the quality of administrative data in relation to the use these sections (Care Quality Commission, 2014).

### Exposures

The MHMDS contains limited socio-demographic patient data for each patient, but some variables had levels of missingness too high for inclusion in statistical models (marital status, 15% missingness; accommodation status, 64% missingness; employment status, 75% missingness and psychiatric diagnosis, 81% missingness). Thus, analyses were restricted to three individual-level exposures: (1) gender (male/female); (2) age at the start of the reporting period (range 0–114 years, median age 48 years – categorised in four age group bands <18 years, 18–35 years, 36–64 years, 65+ years) and (3) ethnicity, five composite groups as per Census 2011: *White British*, *White other* (white Irish or any other white background), *Black or Black British* (Caribbean, African, any other black background), *Asian or Asian British* (Indian, Pakistani, Bangladeshi, Chinese or any other Asian background), *Mixed* (white and black Caribbean, white and black African, white and Asian or any other mixed background) and *any other* ethnic group.

As operationalised elsewhere (Bécares & Das-Munshi, 2013), own-group ethnic density was calculated from 2011 Census data for each LSOA, with the number of residents from an ethnic group in each area divided by the total population of the area; this was calculated for all individuals in each ethnic group (White other, Black, Asian and mixed) and for White British individuals. For each ethnic group, the own-group ethnic density variable was divided by 10 to show the odds of experiencing compulsory admission per a 10% increase in own-group ethnic density and was added to the multilevel model as a continuous (grand mean centred) variable.

The 2010 IMD (Department for Communities and Local Government, 2011) is based on 38 separate indicators recorded at the LSOA level reflecting seven domains of deprivation (income, employment, health, education, barriers to housing and services, crime and living environment). The IMD summary score for each LSOA was used and divided into quintiles, with the least deprived quintile serving as the reference category. No NHS Provider Trust level explanatory variables were included in the statistical models as they had been found to have limited explanatory power previously (Weich et al., 2017).

Population density (number of people per hectare) was calculated for each LSOA and divided into quintiles, with the least populated quintile serving as the reference category.

### Data preparation and the analytical sample

Excluded from the study sample were patients who had missing data for gender (*n* = 454; <0.001%), age (*n* = 84; <0.001%), ethnicity (118 091; 9.6%) and LSOA identifier (11 479; 0.1%). Patients with missing ethnicity data were characterised as follows: 55.8% were female, 37.9% were aged 36–64 years, 0.8% were detained under MHA during the 2010–2011 reporting period, 22.7% living in most deprived areas and 21.9% lived in most densely populated areas. Patients classified as ‘any other ethnic group’ (19 916; 1.6%) were also excluded because it was not possible to calculate own-group ethnic density from Census data for this group. Listwise deletion of missing data resulted in an analytic sample of 1 053 617 (85.1% of study sample).

### Statistical analyses

Multilevel logistic regression models were conducted taking account of the clustering of patients within both LSOAs and NHS Trusts. As the relationship between LSOAs and NHS Trusts is complex, with patients from the same LSOA receiving care from different Trusts and each Trust covering a large number of LSOAs, cross-classified multilevel models were used (Fielding & Goldstein, 2006; Weich et al., 2017).

A model building process was conducted. First, five models were estimated, one for each ethnic group (models 1a–1e) to test the overall effect of own-group ethnic density (mean level/LSOA) on risk of compulsory admission, controlling only for patient gender (females *v.* males) and age (<18 years *v.* 18–35 years, 36–64 years and 65+ years groups). Models 2a–2e added IMD to assess the strength of the association between ethnic density and risk of compulsory admission while controlling for area-level deprivation, gender and age. Models 3a–3e were estimated to extend models 2a–2e to further control for area-level population density. Supplementary analyses were also conducted to re-estimate models 2a–2e replacing IMD with area-level population density to examine for autocorrelation and confounding between IMD and population density (see online Supplementary Table S1, models 4a–4e).

All models were estimated using MLwiN (Rasbash, Charlton, Jones, & Pillinger, 2017) using Bayesian Markov chain Monte Carlo (MCMC) estimation methods, initially with burn-in length of 500, following by up to 1M iterations to ensure stable model estimates, determined by standard diagnostic measures for MCMC models. Odds ratios [ORs; 95% credible intervals (CI)] are reported accompanied by Bayesian *p* values. Consideration was given to the size of effect of the ORs in interpreting the model results (as per Chen, Cohen, and Chen (2010); <1.68 = very small, 1.68–3.46 = small, 3.47–6.71 = medium; >6.71 = large). The effects for own-group ethnic density are interpreted first, followed by the effect sizes for covariates in the model. The goodness of fit of alternative models was compared using the Bayesian deviance information criterion (DIC) (Spiegelhalter, Best, Carlin, & Van Der Linde, 2002), which is considered analogous to the Bayesian information criterion (Schwarz, 1978) or the Akaike Information Criterion (Akaike, 1987) fit statistics for models estimating using MCMC. Models with the smallest DIC values are considered the best model fit, with a difference of 10 or more considered to be substantial.

## Results

### Sample characteristics

Table 1 presents the socio-demographic characteristics of the analytic sample. Statistically significant differences in the distribution of age, gender, compulsory admission, area-level deprivation and population density were evident across the ethnic groups. Specifically, Black patients had the highest percentage of compulsory admission (12.4%), followed by mixed-ethnicity patients (7.3%), then Asian patients (5.9%), then White other (4.5%), with White British patients having the lowest (3.0%). Although the distribution of White British and White other patients was spread almost equally across the five IMD quintiles, over one-third of Black and Asian patients, and over one-quarter of mixed-ethnicity patients, lived in the most deprived areas. Mean levels of own-group ethnic density (calculated separately for each ethnic group) were highest for White British patients, followed by Asians, Black, White other and mixed-ethnicity.

**Table 1.** Descriptive information of the outcome measures and independent variables for the five composite ethnic patient groups

Patient/area-level characteristic	White British (*n* = 894 519; 84.9%)	White other (*n* = 63 640; 6.0%)	Black (*n* = 36 480; 3.5%)	Asian (*n* = 46 076; 4.4%)	Mixed (*n* = 12 902; 1.2%)	χ^2^ (df)
Treatment						
Compulsory admission	27 256 (3.0%)	2847 (4 ⋅5%)	4536 (12.4%)	2741 (5.9%)	940 (7.3%)	10 263.796 (4) *p* < 0.0001
Other	867 263 (97.0%)	60 793 (95 ⋅5%)	31 944 (87.6%)	41 545 (94.1%)	11 962 (92.7%)	
Gender						
Male	390 432 (43.6%)	27 716 (43.6%)	18 260 (50.1%)	22 574 (49.0%)	5987 (46.4%)	1081.614 (4) *p* < 0.0001
Female	504 087 (56.4%)	35 924 (56.4%)	18 220 (49.9%)	23 502 (51.0%)	6915 (53.6%)	
Age						
<18 years	14 984 (1.7%)	660 (1.0%)	476 (1.3%)	634 (1.4%)	487 (3.8%)	22 099.977 (12) *p* < 0.0001
18–35 years	210 528 (23.5%)	16 353 (25.7%)	12 892 (35.3%)	16 858 (38.2%)	6122 (47.5%)	
36–64 years	356 864 (39.9%)	27 124 (42.6%)	18 162 (49.8%)	20 261 (45.9%)	5056 (39.2%)	
65+ years	312 143 (34.9%)	19 503 (30.6%)	4950 (13.6%)	6381 (14.5%)	1237 (9.6%)	
Own-group ethnic density, *M* (s.d.) [Range]	84.17 (15.044) [1–100]	10.49 (8.340) [0–50]	17.18 (13.102) [0–64]	33.16 (25.226) [0–99]	3.97 (2.418) [0–14]	NA
Index of multiple deprivation (IMD)						
First quintile (least deprived)	188 774 (21.1%)	9434 (14.8%)	1375 (3.8%)	4279 (10.9%)	1412 (10.9%)	29 493.212 (16) *p* < 0.0001
Second	188 649 (21.1%)	11 111 (17.5%)	2871 (7.9%)	6203 (13.5%)	1969 (15.3%)	
Third	180 032 (20.1%)	13 058 (20.5%)	6501 (17.8%)	8971 (19.5%)	2561 (19.8%)	
Fourth	168 974 (18.9%)	16 421 (25.8%)	12 650 (34.7%)	11 847 (25.7%)	3399 (26.3%)	
Fifth (most deprived)	168 090 (18.8%)	13 616 (21.4%)	13 083 (35.9%)	14 776 (32.1%)	3561 (27.6%)	
Population density						
First quintile (least populated)	147 156 (16.5%)	4805 (7.6%)	666 (1.8%)	1139 (2.5%)	728 (5.6%)	90 721.673 (16) *p* < 0.0001
Second	181 651 (20.3%)	8007 (12.6%)	2455 (6.7%)	4443 (9.6%)	1583 (12.3%)	
Third	196 623 (22.0%)	10 314 (16.2%)	3865 (10.6%)	6614 (14.4%)	2151 (16.7%)	
Fourth	201 567 (22.5%)	12 832 (20.2%)	5867 (16.1%)	9625 (20.9%)	2738 (21.2%)	
Fifth (most populated)	167 522 (18.7%)	27 682 (43.5%)	23 627 (64.8%)	24 255 (52.6%)	5702 (44.2%)	

χ^2^, chi-square test; df, degrees of freedom.

Data from the 2010–2011 Mental Health Minimum Dataset (*N* = 1 053 617).

### Ethnic density effects: by ethnic group

A mixed pattern was observed for the association between own-group ethnic density and the odds of compulsory admission (see Table 2). Only the results for patients of White British, White other and mixed-ethnicity were statistically significant. For White British patients, a 10% increase in own-group ethnic density was associated with a protective effect against the risk of compulsory admission (OR 0.93, 95% CI 0.92–0.94) whereas for patients of White other or mixed ethnicity, the opposite trend emerged – a 10% increase in own-group ethnic density was associated with a statistically significant increased risk in the odds of compulsory admission (White other: OR 1.21, 95% CI 1.13–1.29; mixed: OR 1.64, 95% 1.11–2.41).

**Table 2.** Risk of compulsory admission estimated separately by ethnic group, accounting for patient-level sex, age and ethnic density, derived from 3-level (patients within LSOAs within NHS Provider Trust) cross-classified multi-level model

		OR (95% CIs)			
	White British (*n* = 894 519) Model 1a	White Other (*n* = 63 640) Model 1b	Black (*n* = 36 480) Model 1c	Asian (*n* = 46 076) Model 1d	Mixed (*n* = 12 902) Model 1e
Cons	−4.117 (0.111)	−3.263 (0.176)	−2.993 (0.187)	−3.781 (0.252)	−4.248 (0.291)
*Level 1* (*Patient*)					
Gender					
Women (Ref.)					
Men	1.23 (1.20–1.26)***	1.24 (1.15–1.34)***	1.44 (1.35–1.54)***	1.53 (1.42–1.67)***	2.03 (1.74–2.36)***
Age					
Under 18 years (Ref.)					
18–35 years	1.69 (1.20–1.90)***	1.14 (0.82–1.60)	2.42 (1.72–3.40)***	2.58 (1.60–4.15)***	2.87 (1.71–4.84)***
36–64 years	1.79 (1.60–2.00)***	1.02 (0.73–1.43)	1.66 (1.18–2.34)**	1.77 (1.10–2.85)*	2.32 (1.37–3.92)***
65 years and older	1.08 (0.96–1.21)***	0.69 (0.49–0.97)***	0.78 (0.55–1.22)	0.80 (0.49–1.31)	1.20 (0.66–2.19)
*Level 2* (*LSOA*)					
Own- group Ethnic density	0.93 (0.92–0.94)***	1.21 (1.13–1.29)***	1.01 (0.98–1.05)	0.98 (0.96–1.01)	1.64 (1.11–2.41)*

Data from the 2010–2011 Mental Health Minimum Dataset (*N* = 1 053 617).

Bayesian *p* value: ***<0.001, **<0.01, *<0.05.

Overall, the trend was for a very small to small, but statistically significant, increased risk of compulsory admission among men across the ethnic groups (smallest effect sizes for the White groups, largest for the mixed ethnic group). Younger patients (aged 18–35 years) in the Black, Asian and mixed ethnic groups experienced a small increased risk of compulsory admission, but this effect size decreased as age increased. For the White British group, the small increased risk of compulsory admission was evident for two middle age groups.

### Ethnic density effects, controlling for deprivation: by ethnic group

The inclusion of area-level deprivation (Table 3) changed the overall picture somewhat. White British patients continued to experience a decrease in the odds of compulsory admission with each 10% increase in own-group ethnic density (OR 0.94, 95% CI 0.93–0.95), whereas White other patients continued to experience an increase in the odds of compulsory admission (OR 1.18, 95% CI 1.11–1.26) (models 2a and 2b, respectively). For patients of mixed-ethnicity, the previously observed increase in the odds of compulsory admission when living in the areas of higher own-group ethnic density (model 1e), was non-statistically significant when area-level deprivation was included in the model (model 2e). The effect of own-group ethnicity for Asian patients now emerged as statistically significant, meaning that a 10% increase in ethnic density was associated with a protective effect against compulsory admission but only while accounting for area-level deprivation. For the Black group, there was no statistically significant association between own-group ethnic density and compulsory admission when IMD was included in the model (model 2b).

**Table 3.** Risk of compulsory admission estimated separately by ethnic group, accounting for patient-level sex, age, ethnic density and area-level deprivation, derived from 3-level (patients within LSOAs within NHS Provider Trust) cross-classified multi-level model

		OR (95% CIs)			
	White British (*n* = 894 519) Model 2a	White Other (*n* = 63 640) Model 2b	Black (*n* = 36 480) Model 2c	Asian (*n* = 46 076) Model 2d	Mixed (*n* = 12 902) Model 2e
Cons	−4.312 (0.072)	−3.556 (0.166)	−3.161 (0.212)	−4.194 (0.216)	−4.518 (0.339)
*Level 1* (*Patient*)					
Gender					
Women (Ref.)					
Men	1.23 (1.20–1.26)***	1.23 (1.14–1.33)***	1.44 (1.35–1.54)***	1.53 (1.41–1.65)***	2.02 (1.74–2.35)***
Age					
Under 18 years (Ref.)					
18–35 years	1.72 (1.52–1.94)***	1.18 (0.85–1.64)	2.39 (1.69–3.39)***	2.73 (1.81–4.12)***	2.95 (1.68–5.25)***
36–64 years	1.82 (1.61–2.05)***	1.06 (0.76–1.47)	1.64 (1.16–2.33)**	1.89 (1.25–2.84)***	2.38 (1.36–4.20)**
65 years and older	1.11 (0.98–1.25)	0.73 (0.52–1.02)	0.77 (0.54–1.12)	0.87 (0.57–1.34)	1.25 (0.66–2.36)
*Level 2* (*LSOA*)					
Own-group ethnic density	0.94 (0.93–0.95)***	1.18 (1.11–1.26)***	1.02 (0.98–1.06)	0.97 (0.95–0.99)**	1.40 (0.91–2.15)
IMD					
First quintile (least deprived) (Ref.)					
Second quintile	1.12 (1.07–1.18)***	1.28 (1.11–1.49)***	1.28 (1.02–1.61)*	1.25 (1.05–1.49)*	1.14 (0.83–1.57)
Third quintile	1.20 (1.14–1.26)***	1.35 (1.16–1.57)***	1.29 (1.04–1.54)*	1.42 (1.21–1.67)***	1.33 (0.98–1.82)
Fourth quintile	1.20 (1.14–1.27)***	1.32 (1.14–1.53)***	1.19 (0.96–1.47)	1.40 (1.19–1.63)***	1.26 (0.92–1.72)
Fifth quintile (most deprived)	1.21 (1.15–1.28)***	1.41 (1.21–1.64)***	1.18 (0.95–1.48)	1.63 (1.37–1.93)***	1.36 (0.99–1.89)

Data from the 2010–2011 Mental Health Minimum Dataset (*N* = 1 053 617).

Bayesian *p* value: ***<0.001, **<0.01, *<0.05.

In models 2a–2e, an overall trend emerged for a statistically significant increased odds of compulsory admission when living in the areas of higher deprivation for White British, White other and Asian patients. For Black patients, only the effects for the second and third quintiles were statistically significant (model 2c), whereas for mixed patients, the effects for IMD were not statistically significant (model 2e). Overall, the trends for gender and age remained constant.

### Ethnic density effects, controlling for deprivation and population density: by ethnic group

The addition of population density in the final stage of analysis (models 3a–3e; Table 4) did not change the model results. Supplementary analyses (models 4a–4e; online Supplementary Table S1) were conducted to examine the effect of population density when IMD was excluded. The effects of both IMD and population density were similar when both variables were included in models together (as shown between Tables 3 and 4 for IMD) and between Table 4 and online Supplementary Table S1 for population density.

**Table 4.** Risk of compulsory admission estimated separately by ethnic group, accounting for patient-level sex, age, area deprivation and population density, derived from 3-level (patients within LSOAs within NHS Provider Trust) cross-classified multi-level model

		OR (95% CIs)			
	White British (*n* = 894 519) Model 3a	White Other (*n* = 63 640) Model 3b	Black (*n* = 36 480) Model 3c	Asian (*n* = 46 076) Model 3d	Mixed (*n* = 12 902) Model 3e
Cons	−4.400 (0.009)	−3.570 (0.219)	−3.402 (0.263)	−3.977 (0.300)	−4.474 (0.380)
*Level 1* (*Patient*)					
Gender					
Women (Ref.)					
Men	1.23 (1.20–1.26)***	1.23 (1.14–1.33)***	1.44 (1.35–1.54)***	1.53 (1.41–1.65)***	2.02 (1.74–2.36)***
Age					
Under 18 years (Ref.)					
18–35 years	1.78 (1.57–2.00)***	1.14 (0.78–1.67)	2.39 (1.69–3.56)***	2.62 (1.69–4.31)***	2.98 (1.74–5.37)***
36–64 years	1.88 (1.66–2.11)***	1.02 (0.78–1.67)	1.64 (1.17–2.45)**	1.80 (1.16–2.97)***	2.41 (1.41–4.37)***
65 years and older	1.15 (1.01–1.29)*	0.70 (0.48–1.03)	0.77 (0.54–1.12)	0.83 (0.53–1.40)	1.26 (0.67–2.44)
*Level 2* (*LSOA*)					
Own-group ethnic density	0.94 (0.93–0.95)***	1.19 (1.11–1.28)***	1.03 (0.99–1.07)	0.97 (0.95–1.00)*	1.45 (0.93–2.28)
IMD					
First quintile (least deprived) (Ref.)					
Second quintile	1.12 (1.07–1.17)***	1.28 (1.10–1.50)***	1.30 (1.04–1.64)*	1.25 (1.03–1.53)*	1.14 (0.82–1.60)
Third quintile	1.20 (1.13–1.26)***	1.34 (1.16–1.57)***	1.30 (1.05–1.62)*	1.43 (1.19–1.73)***	1.35 (0.98–1.87)
Fourth quintile	1.20 (1.14–1.26)***	1.32 (1.13–1.55)***	1.20 (0.97–1.50)	1.41 (1.17–1.71)***	1.28 (0.93–1.78)
Fifth quintile (most deprived)	1.20 (1.13–1.26)***	1.41 (1.20–1.67)***	1.19 (0.96–1.50)	1.65 (1.36–2.00)***	1.39 (0.93–1.96)
Population density					
First quintile (least populated) (Ref.)					
Second quintile	1.05 (1.00–1.11)*	1.04 (0.85–1.28)	1.48 (1.08–2.03)**	0.87 (0.64–1.19)	0.98 (0.64–1.52)
Third quintile	1.08 (1.02–1.14)*	1.13 (0.93–1.37)	1.32 (0.97–1.81)*	0.85 (0.64–1.16)	0.88 (0.58–1.34)
Fourth quintile	1.04 (0.99–1.10)	1.08 (0.89–1.31)	1.30 (0.96–1.76)	0.82 (0.61–1.11)	0.95 (0.64–1.45)
Fifth quintile (most populated)	1.05 (0.99–1.11)	1.02 (0.84–1.24)	1.19 (0.89–1.63)	0.83 (0.63–1.13)	0.90 (0.59–1.37)

Data from the 2010–2011 Mental Health Minimum Dataset (*N* = 1 053 617).

Bayesian *p* value: ***<0.001, **<0.01, *<0.05.

### Model comparison: DIC

Table 5 presents the goodness of fit for the three sets of models: ethnic density, age and gender only (models 1a–1e); additionally, controlling for area-level deprivation (models 2a–2e); and additionally, controlling for population density (models 3a–3e). The model fit for the supplementary analyses (models 4a–4e, population density excluding IMD) are also presented. Models with the lowest values indicate best model fit to the data. For the White British, White other and Asian groups, models 2a–2e (which included area-level deprivation), had the lowest DIC values by at least 10 points compared to the models containing own-group ethnic density only. For the Black and mixed-ethnic groups, none of the models differed by 10 points; for the Black group, model 1c with own-group ethnic density had the lowest DIC value, whereas for the mixed-ethnicity group, model 2e had the lowest DIC value. Overall, the DIC statistics indicated that models with IMD alone were the most parsimonious models.

**Table 5.** Goodness of fit, as measured by the Bayesian deviance information criterion (DIC), obtained from multilevel cross-classified models (MCMC)

DIC	Ethnic group				
	White British	White other	Black	Asian	Mixed-ethnicity
Models 1a–1e (own-group ethnic density, age, gender)	233 230.72	22 646.31	**26 340.84**	19 875.74	6347.08
Models 2a–2e (model 1, controlling for area-level deprivation only)	**233 143.68**	**22 634.85**	26 343.31	**19 857.06**	**6343.57**
Models 3a–3e (model 2, controlling for population density)	233 154.63	22 639.17	26 342.97	19 862.45	6346.91
Models 4a–4e (model 1, controlling for population density only)	233 214.31	22 648.52	26 340.90	19 881.20	6358.661

Bold significance the lowest value of the DIC, which is indicative of the best model fit.

## Discussion

In this study, we sought to test empirically the ethnic density hypothesis as it relates to the risk of compulsory admission to psychiatric hospitals under specific sections of the MHA in England for 1.2 million patients who received treatment provided by NHS-funded mental health services during 2010–2011. Our focus on characterising own-group ethnic density at the neighbourhood level advances previous studies which have found mixed evidence of an ethnic density effect on risk of compulsory admission when ethnic density was characterised at higher spatial levels.

Summarising our findings succinctly, we found limited evidence in support of the ethnic density hypothesis i.e. living in neighbourhoods characterised by high own-group ethnic density level offered a small, protective buffer against the risk of compulsory admission for a few minority ethnic groups we studied. Asian patients experienced a very small protective effect (3% reduction), but only after accounting for area-level deprivation. White British patients experienced a small (7%) reduction, which remained stable after adjusting for area-level deprivation and population density. In contrast, and running counter to the ethnic density hypothesis, patients of White minority ethnicity experienced a small (21%) increased risk of the odds of compulsory admission and this effect remained after accounting for area-level deprivation and population density. For Black patients, a consistent non-statistically significant effect of own-group ethnic density on compulsory admission emerged in all models suggesting that, for Black patients in the 2010–2011 MHMDS, area-level ethnic density composition had little impact on the odds of compulsory admission. For patients of mixed-ethnicity, the small increase in the odds of compulsory admission was no longer statistically significant after accounting for area-level deprivation or population density. Overall, our findings indicate that, for the different ethnic groups we studied, the effect of ethnic density operates differently to influence the risk of compulsory admission.

Before interrogating these findings, we highlight key strengths and limitations of this study. The major strength of this research lies in the national representativeness of the MHMDS, which contains treatment records for all individuals who received secondary care mental health services in England during 2010–2011. The MHMDS reflects the complex, real-world settings in which patients live and receive mental health services. The MHMDS afforded sufficiently large numbers of Black and minority ethnic patients to conduct meaningful stratified analyses, which has been noted as a weakness in similar-type studies (Shaw et al., 2012). Moreover, the availability of patient-level spatial identifiers permitted linkage to external data sources containing measures of area-level ethnic density and deprivation, which permitted the derivation of an appropriate own-group ethnic density indicator. The use of multilevel models, which could handle the complex structure of the MHMDS, permitted an estimation of the risk of compulsory psychiatric admission across a highly complex geography of places and health care providers; this means our results are highly generalisable to England as a whole.

A major limitation of the study is that the MHMDS includes data of variable quality and completeness. As we have detailed elsewhere (Weich et al., 2014), it was methodologically challenging to analyse the 2010–2011 MHMDS because researchers have to decipher and collate information from multiple variables in the dataset (e.g. number of days spent in hospital/admissions and discharges/consultant episodes/outpatient attendances; and least and most legal restrictive MHA classification status applied to the patient's health care record), to determine whether a patient was compulsory admitted, or not, during the reporting period (i.e. our core study outcome). Given that we were unable to ascertain the number and duration of episodes of compulsory admission, it was not possible to determine whether the patients were experiencing their first or subsequent admissions. Re-admissions are likely to be driven, in part, by patients experiencing more chronic and/or severe mental health difficulties. We were also unable to examine ethnic differences in terms of how different sections of the MHA were applied to patients' care records and we acknowledge that there are plausible reasons as to why this is an important consideration when examining ethnic differences in outcomes relating to use of the MHA. For example, sections of the MHA involving conveyance to, or assessment in, registered Places of Safety, including police stations, might be administered differently across ethnic groups. We excluded these patients because use of these sections do not necessarily mean that the individual will be admitted to an inpatient mental health bed; for example, a study conducted in Gloucestershire during 2002–2006 suggested that ~30% of individuals detained under S136 were subsequently admitted to hospital (Laidlaw, Pugh, Riley, & Hovey, 2010). Also, there is evidence of ongoing, wide variation between regions in England with regard to the use of hospitals or police stations as Places of Safety and this may be explained, in part, by different recording practices used by police forces (Care Quality Commission, 2014; Keown, 2013).

The MHMDS often lacks information regarding reasons for some compulsorily admissions, such as insights into the patient's mental health history, as well of aspects of their family, community and social networks. Even for data that were recorded, high levels of missing data were evident on key variables of interest, and we believe that missing data on psychiatric diagnosis (~80%) and ethnicity (~10%) to be most relevant to our study implications. Specifically, we were unable to test whether there was evidence of an ethnic density effect for different psychiatric disorders which resulted in compulsory admission, even though the existing literature suggests that the most robust evidence for the hypothesis might emerge for psychotic-related disorders. Moreover, it was not clear from the MHMDS specification documents why there was such a high level of missing data for patient ethnicity. In our previous study (Weich et al., 2014), we considered that although it might be possible that patients from certain ethnic groups preferred not to declare their ethnicity (e.g.) because they believed that it would affect their treatment, a more likely explanation is that NHS Trusts with little ethnic diversity among its patient population were less rigorous in identifying and recording this information. As highlighted previously, <1% of this group experienced detention under the MHA and so we argue that excluding this group of individuals should not have had substantial impact on our findings. Despite the large size of the MHMDS, we acknowledge that our study may have lacked the power to detect true variation in compulsory admission for some groups (e.g. mixed ethnicity group).

Notwithstanding these limitations, our findings meaningfully contribute to the evidence base by demonstrating that ethnic density effect operates differently at the neighbourhood level for specific ethnic groups to influence (both increase and decrease) the risk of compulsory admission to psychiatric hospital. The reasons for the reduced risk of compulsory admission may point to different mechanisms operating at the community level. For example, the buffer experienced by the White British majority is consistent with earlier findings of the protective effect of high levels of ethnic concentration (Wechsler & Pugh, 1967), which suggest that White British patients when living in areas largely characterised by their own group ‘fit’ with their communities and appear to benefit from this living environment, regardless of neighbourhood level deprivation or population density. An alternative hypothesis is that the areas with higher rates of White British ethnicity are often in rural areas, and in these rural areas rates of psychosis are lower. It may be that if accurate diagnostic information was available this may have impacted the model. Similarly, our findings in relation to White minority patients, largely concur with those reported by Terhune et al. (2020) in Sweden: higher rates of compulsory admission among migrants in Sweden was associated with living in neighbourhoods with higher-rates of migrant density, which suggests that there may be concentrated cultural factors which affect timely access to mental health treatment services in these local communities.

The observed protective effect in this study for Asian patients is less easy to interpret. For example, it is recognised that individuals of South Asian ethnicity appear to experience mental health difficulties differently to other minority communities (Bhui & Bhugra, 2002; Time to Change, 2010), and it is not clear from this study whether the small reduced risk of compulsory admission linked to living in areas of high own group ethnic density relates to reduction in mental distress experienced by the individual because of support provided by that neighbourhood (e.g. close family ties providing support), or whether the higher ethnic concentration at the neighbourhood level acts as a barrier to seeking secondary care mental health services more generally (e.g. through feelings of shame relating to cultural interpretations of mental illness).

If replicated, our findings have implications for Providers and Commissioners of secondary care mental health services who oversee administration of the MHA in England. The take-home message is that stakeholders need to be more mindful of the nature or characteristics of places in which the patients in their local catchment area live and experience symptoms of mental disorders. This is particularly relevant given the final report of the 2018 Independent Review of the MHA (Department of Health and Social Care, 2018) advocated strongly for a reform of secondary care mental health service provision and the delivery of mental health treatment in the least restrictive environment possible. Also, a key recommendation of the Independent Review is that future research studies are co-produced with the input of service users, carers and communities to fully understand, and ultimately effectively address, the persistent and substantial ethnic inequalities in compulsory mental health treatment outcomes in England. Although this study supports these aims, there is a need for specifically designed research protocols which examine the characteristics of the areas in which ethnic minority patients live, how patients from different ethnic groups feel about their neighbourhoods, and how they use (or otherwise) the community supports available to them in these areas during periods when they experience mental health difficulties. Only through exploring these issues in more detail will it be possible to develop a greater understanding of how patients from different ethnic backgrounds interact with, and progress through, the complex system of secondary care mental health services in England.

## Supplementary material

For supplementary material accompanying this paper visit https://doi.org/10.1017/S0033291721001768. click here to view supplementary material
