# Supplementary material for: Effects of ethnic density on the risk of compulsory psychiatric admission for individuals attending secondary care mental health services: evidence from a large-scale study in England
Source: Psychol Med. 2021 May 20;53(2):458–67. doi: 10.1017/S0033291721001768 (PMC9899561; doi:10.1017/S0033291721001768)
Supplement: Supplementary file 1 [file S0033291721001768sup001.docx]

Table S1 Risk of compulsory admission estimated separately by ethnic group, accounting for patient-level sex, age, and population density, derived from 3-level (patients within LSOAs within NHS Provider Trust) cross-classified multi-level model. Data from the 2010-11 Mental Health Minimum Dataset (N=1,053,617).

|  | | |  | Odds ratio (95% Credible Intervals) | | | |
| --- | --- | --- | --- | --- | --- | --- | --- |
|  |  |  | White British  (n=894519)  **Model 3a** | White Other  (n=63640)  **Model 3b** | Black  (n=36,480)  **Model 3c** | Asian  (n=46,076)  **Model 3d** | Mixed  (n=12,902)  **Model 3e** |
| Cons |  |  | -4·32 (0·082) | -3·388 (0·213) | -3·402 (0·263) | -3.727 (0·281) | -4.204 (0·325) |
| Level 1 (Patient) | Gender | Women (Ref.) |  |  |  |  |  |
|  |  | Men | 1·23 (1·20-1·26)*** | 1·24 (1·15-1·34)*** | 1·45 (1·35-1·55)*** | 1·53 (1·42-1·66)*** | 2·03 (1·75-2·35)*** |
|  | Age | Under 18 years (Ref.) |  |  |  |  |  |
|  |  | 18-35 years | 1·80 (1·59-2.04)*** | 1·14 (0·80-1·66) | 2·53 (1·78-3·80)*** | 2·53 (1·62-3.99)*** | 2·85 (1·75-4.79)*** |
|  |  | 36-64 years | 1·90 (1·69-2·16)*** | 1·02 (0·72-1·49) | 1·74 (1·22-2·62)*** | 1·74 (1·11-2·74)** | 2·31 (1·68-3.93)*** |
|  |  | 65 years and older | 1·15 (1.02-1·30)* | 0·70 (0·48-1·00)* | 0·82 (0·57-1·24) | 0·79 (0·49-1·27) | 1·20 (0·66-2·17)** |
| Level 2  (LSOA) | Own-group ethnic density | | 0·93 (0·92-0·94)*** | 1·21 (1·12-1·29)*** | 1·02 (0·98-1·05) | 0·98 (0·96-1.01) | 1·66 (1.10-2·47)** |
|  | Population density | first quintile (least populated) (Ref.) |  |  |  |  |  |
|  |  | second quintile | 1.07 (1.02-1.13)*** | 1.08 (0.89-1.32) | 1.48 (1.08-2.00)** | 0.94 (0.71-1.26) | 1.02 (0.68-1.54) |
|  |  | third quintile | 1.11 (1.06-1.17)*** | 1.19 (0.98-1.44) | 1.33 (0.98-1.77)* | 0.94 (0.71-1.25) | 0.92 (0.62-1.37) |
|  |  | fourth quintile | 1.09 (1.04-1.15)*** | 1.16 (0.97-1.40) | 1.31 (0.97-1.73)* | 0.92 (0.70-1.22) | 1.01 (0.62-1.37) |
|  |  | fifth quintile (most populated) | 1.10 (1.04-1.17)*** | 1.13 (0.93-1.36) | 1.21 (0.90-1.59) | 0.98 (0.75-1.29) | 0.96 (0.65-1.42) |

Bayesian p-value: ***<0.001, **<0.01, *<0.05
